# Antioxidant capacity of chewing stick miswak *Salvadora persica*

**DOI:** 10.1186/1472-6882-13-40

**Published:** 2013-02-21

**Authors:** Saleh A Mohamed, Jalaluddin A Khan

**Affiliations:** 1 Biochemistry Department, Faculty of Science, King Abdulaziz University, Jeddah, 21589, Kingdom of Saudi Arabia

**Keywords:** Miswak, Root, Furan Derivatives, Antioxidant Activities

## Abstract

**Background:**

Chewing stick (miswak *Salvadora persica* L.) is an effective tool for oral hygiene. It possessed various biological properties including significant antibacterial and anti-fungal effects. In the present study, we evaluated the antioxidant compounds in miswak.

**Method:**

Miswak root was extracted with 80% methanol. Methanol extract as antioxidant was evaluated by using DPPH, ABTS and phosphomolybdenum complex assays and analysis by GC-MS. Peroxidase, catalase and polyphenoloxidase assays were performed for crude extract of miswak root.

**Results:**

The methanol extract of miswak contained the highest amount of crude extract among the various solvent extracts. The methanol extract showed a concentration dependent scavenging of DPPH and ABTS radicals with IC_50_ values 4.8 and 1.6 μg crude extract, respectively. The total antioxidant activities, based on the reduction of molybdenum (VI) to molybdenum (V), increased with increasing crude extract content. The correlation coefficients (*R*^2^) between total crude extract and DPPH, ABTS scavenging activities and the formation of phosphomolybdenum complex were 0.97, 0.99 and 0.95, respectively. The GC-MS analysis showed that the methanol extract doesn’t contain phenolic and flavonoid compounds or under detected limit. After silylation of methanol extract, three compounds namely 2-furancarboxaldehyde-5-(hydroxymethyl), furan-2-carboxylic acid-3-methyl- trimethylsilyl ester and D-erythro-pentofuranose-2-deoxy-1,3,5-tris-O-(trimethylsilyl) were identified by GC-MS analysis. These furan derivatives as they contain hydroxyl groups could be possessed antioxidant activities. The antioxidant enzymes were also detected in the miswak extract with high level of peroxidase and low level of catalase and polyphenoloxidase.

**Conclusions:**

The synergistic actions of antioxidant compounds and antioxidant enzymes make miswak is a good chewing stick for oral hygiene and food purposes.

## Background

The use of the chewing stick (miswak) for cleaning teeth is an ancient custom which remains widespread in many parts of the world [1,2]. The World Health Organization has recommended and encouraged the use of chewing sticks as an effective tool for oral hygiene in areas where such use is customary [3]. Among at least 182 plant species suitable for preparing toothbrushing sticks, miswak harvested from *Salvadora persica* (family name: Salvadoraceae), are used most extensively [4]. The roots, twigs, and stems of this miswak have been used for oral hygiene [5] and small *S. persica* sticks have been used as toothpicks [6]. It has been shown that extracts of miswak posses various biological properties including significant antibacterial [7] and anti-fungal effects [8]. Extracts of *S. persica* and other related plants may be effective against the bacteria that are important for the development of dental plaque. Therefore, it has been claimed that miswak sticks may have antiplaque effects and may also affect the pathogenesis of periodontal diseases by reducing the virulence of periodontophathogenic bacteria [9]. Almas [10] reported that miswak and chlorahexidine gluconate had the same effect on healthy human dentin. The anti-microbial and cleaning effects of miswak have been attributed to various chemicals detectable in its extracts such as sodium chloride and potassium chloride as well as salvadourea and salvadorine, saponins, vitamin C, silica and resin [11].

Antioxidants are substances that when present in foods or body at low concentrations compared with that of an oxidizable substrate markedly delay or prevent the oxidation of that substrate. The antioxidants included enzymatic antioxidants (e.g., superoxide dismutase, peroxidase, polyphenoloxidase and catalase) and non-enzymatic antioxidants (e.g., ascorbic acid (vitaminC), α-tocopherol (vitamin E), glutathione, carotenoids, and flavonoids) [12]. Antioxidants may help the body to protect itself against various types of oxidative damage caused by reactive oxygen species, which are linked to a variety of diseases including cardiovascular diseases, cancers [13], neurodegenerative diseases, Alzheimer’s disease [14] and inflammatory diseases [15]. The supplement of the diet (or other uses) with antioxidant compounds is one of solutions of this problem that are contained in natural plant sources [16]. These natural plant antioxidants can therefore serve as a type of preventive medicine. Some researchers suggest that two-thirds of the world’s plant species have medicinal value; in particular, many medicinal plants have great antioxidant potential [12].

Despite several studies had been focused on the chewing stick miswak *Salvadora persica* L. chemical components, which had antimicrobial activity, its bioactive compounds especially antioxidant compounds has not yet been established. Therefore, the antioxidant compounds and antioxidant enzymes of miswak has been studied.

## Methods

### Plant material

Miswak *Salvadora persica* L. (Salvadoraceae) root is wild plant and used as publicly available herbarium. Miswak root was purchased from local market of Jeddah, Kingdom of Saudi Arabia. The identification of miswak is confirmed in voucher sample (Ser. No. 2215) deposited at Herbarium, King Abdulaziz University.

### Chemicals

The solvent used in the present work were purchased from Riedel-de-Haen (Germany). 1,1-Diphenyl-2-picrylhydrazyl (DPPH), 2,2'-azino-bis (3-ethylbenzo-thiazoline-6-sulfonic acid) (ABTS), ammonium molybdate were obtained from Fluka (Germany). Hydrogen peroxide, guaiacol and catechol were purchased from Sigma (USA).

### Preparation of solvent extracts

Dried miswak root (2 g) was extracted by shaking at 150 rpm and 25°C for 24 h with 20 ml of solvents (1:10, w/v) of various polarities including distilled water or methanol (80%) or ethanol (80%) or acetone (80%). The extracts were evaporated in vacuo. The yields of the extracts were recorded.

### DPPH radical scavenging activity

Free radical scavenging activity of crude methanol extract was determined using the 2,2-diphenyl-1-picrylhydrazyl (DPPH) method [17]. A methanol solution (100 μL) containing methanol extracts was added to 900 μL of freshly prepared DPPH methanol solution (0.1 mM). An equal amount of methanol was used as a control. After incubation for 30 min at room temperature in the dark, the absorbance was measured at 517 nm using a spectrophotometer. Activity of scavenging (%) was calculated using the following formula:

$$\begin{array}{l} \text{DPPH}\;\text{radical}\;\text{scavenging}\;\%\;\\ \;=\;\left[\left(\text{OD}\;\text{control}–\text{OD}\;\text{sample}\right)/\text{OD}\;\text{control}\;\right]\;x\;100 \end{array}$$

The results were plotted as the % of scavenging activity against concentration of the sample. The inhibition concentration (IC_50_) was defined as the amount of crude methanol extract required for 50% of free radical scavenging activity. The IC_50_ value was calculated from the plots as the antioxidant concentration required for providing 50% free radical scavenging activity.

### ABTS radical cation decolorization assay

ABTS (2,2'-azino-bis (3-ethylbenzo-thiazoline-6-sulfonic acid) also forms a relatively stable free radical, which decolorizes in its non-radical form. The spectrophotometric analysis of ABTS^•+^ scavenging activity was determined according to the method of Re et al. [18]. ABTS^•+^ was produced by reacting 7 mM ABTS in H_2_O with 2.45 mM potassium persulfate (K_2_S_2_O_8_), store in the dark at room temperature for 16 h. The ABTS^•+^ solution was diluted to give an absorbance of 0.750 ± 0.025 at 734 nm in 0.1 M sodium phosphate buffer (pH 7.4). Then, 1 mL of ABTS^•+^ solution was added to crude methanol extract. The absorbance was recorded 1 min after mixing and the percentage of radical scavenging was calculated relative to a blank containing no scavenger. The extent of decolorization was calculated as percentage reduction of absorbance. The scavenging capability of test compounds was calculated using the following equation:

$$\begin{array}{l} {\text{ABTS}}^{•}{}^{+}\text{scavenging}\;\left(\%\right)\\ \;=\left[\left(\text{OD}\;\text{control}\;–\;\text{OD}\;\text{sample}\right)/\text{OD}\;\text{control}\right]\;x\;100 \end{array}$$

The results were plotted as the % of scavenging activity against concentration of the sample. The inhibition concentration (IC_50_) was defined as the amount of crude methanol extract required for 50% of free radical scavenging activity. The IC_50_ value was calculated from the plots as the antioxidant concentration required for providing 50% free radical scavenging activity.

### Phosphomolybdenum complex assay

Spectrophotometric evaluation of antioxidant activity through the formation of a phosphomolybdenum complex was carried out according to Prieto et al. [19]. Sample solutions (50 μL) were combined in an Eppendorf tube with 1 ml of reagent solution (0.6 M sulfuric acid, 28 mM sodium phosphate and 4 mM ammonium molybdate). The tubes were capped and incubated in a thermal block at 95°C for 90 min. After the samples had cooled to room temperature, the absorbance of aqueous solutions of each was measured at 820 nm against a blank. The antioxidant activity was expressed as the absorbance of the sample.

### GC-MS analysis

The methanol extract of miswak was analyzed by GC–MS Spectrometer (Perkin Elmer, USA) equipped with 30 m x 0.25 mm Elite-1MS column. The carrier gas is helium. The temperature program were set as follows: 50°C hold for 5 min, raised at 10°C /min to 250°C, and hold for 10 min. The injector and detector temperatures were set at 280°C. The ion source and interface temperatures were set at 200 and 250°C, respectively. The mass range was scanned from 50 to 300 amu. The control of the GC–MS system and the data peak processing were controlled by means of Turbo Mass, version 5.4.2.1617 software. Compound identification was verified based on the relative retention time and mass fragmentation pattern spectra with those of standards and the NIST2008. LIB. The silylation of methanol crude extract was performed by trimethylsilyl chloride.

### Determination of antioxidant enzymes

#### Preparation of crude extract

One g of miswak was homogenized with 20 mM Tris–HCl buffer, pH 7.2 contained 0.1 M NaCl using homogenizer. The homogenate was centrifuged at 10,000 rpm for 15 min at 4°C. The supernatant was designed as crude extract and stored at −20°C for further analysis.

### Peroxidase assay

Peroxidase activity was carried out according to Yuan and Jiang [20]. The reaction mixture contained in one ml: 8 mM H_2_O_2_, 40 mM guaiacol, 50 mM sodium acetate buffer, pH 5.5 and least amount of crude extract. The change in absorbance at 470 nm due to guaiacol oxidation was followed for 1 min using a spectrophotometer. One unit of peroxidase activity was defined as the amount of enzyme which increased the O.D. 1.0 per min under standard assay conditions.

### Catalase assay

Catalase activity was determined according to Bergmeyer [21]. Two and half ml of substrate solution was made up of 25 mM H_2_O_2_ in a 75 mM sodium phosphate buffer pH 7.0 and crude extract. The decrease in absorbance at 240 nm and 25°C was recorded for 1 min using a spectrophotometer. One unit of enzyme activity was defined as the amount of the enzyme that causes a change of 0.1 in absorbance per min under standard assay conditions.

### Polyphenoloxidase assay

Polyphenoloxidase activity was assayed with catechol as a substrate according to the spectrophotometric procedure of Jiang et al. [22]. The enzyme solution (100 μL) was rapidly added to 900 μL of 40 mM catechol solution prepared in 0.01 M sodium phosphate buffer, pH 6.8. The increase in absorbance at 400 nm and 25°C was recorded for 3 min using a spectrophotometer. One unit of enzyme activity was defined as the amount of the enzyme that causes a change of 0.1 in absorbance per min.

### Statistical analysis

The statistical analyses were performed by a one-way ANOVA and the Student’s *t*-test. The results were expressed as means ± S.E. to show variations in the various experimental. Difference are considered significant when *P* < 0.05.

## Results and discussion

Extraction of plant by solvent is a commonly used method to obtain antioxidants. However, no single solvent can extract all the antioxidants from plant because of its variation in solubility and polarity [23,24]. In the present study, solvents with different polarities including water, methanol (80%), ethanol (80%) and acetone (80%) were used as solvents to extract antioxidants from of miswak. Table 1 shows the percentage of yields of solvent extracts from dried plant material. The methanol extract contained the highest amount of crude extract.

**Table 1:** Effect of extraction by various solvents on the percentage yields of dried miswak

**Solvent**	**Yield (w/w) (%)**
80% Methanol	1.02 ± 0.054
80% Ethanol	0.62 ± 0.015
80% Acetone	0.702 ± 0.028
Water	0.56 ± 0.008

Values are presented as means ± SE (n = 3).

**Figure 1:**
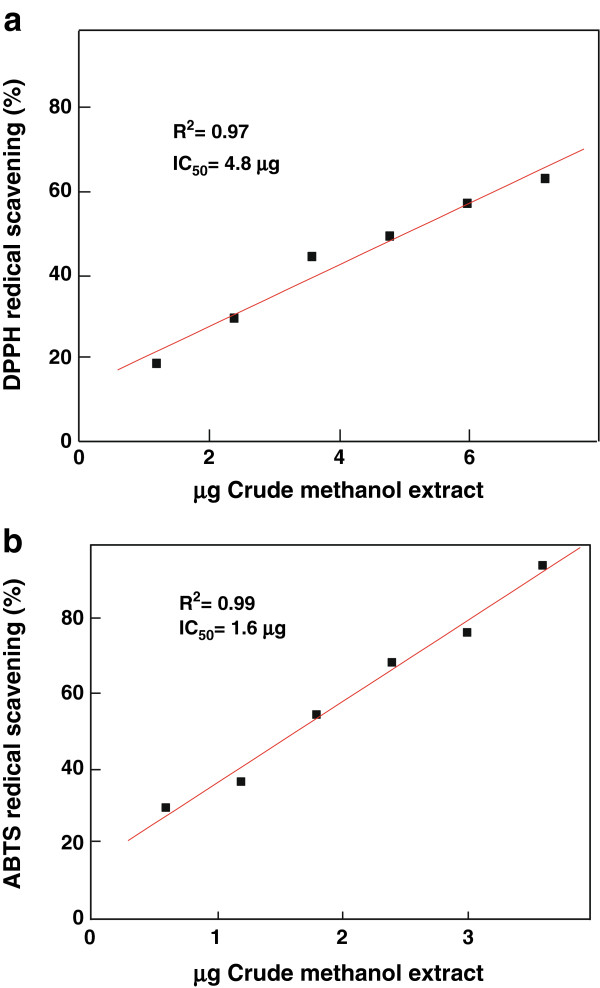
Correlation between different concentrations of miswak crude methanol extracts and their antioxidant capacity as determined by DPPH (a) and by ABTS (b) assays.

The most natural antioxidants are multifunctional. Therefore, a reliable antioxidant evaluation protocol requires different antioxidant activity assessments to take into account various mechanisms of antioxidant action [25]. Therefore, the evaluation of antioxidant activity of methanol extract of miswak was conducted by several methods. Scavenging the stable DPPH radical model is widely used method to evaluate antioxidant activity. The degree of discoloration indicates the scavenging potential of the antioxidant extract, which is due to the hydrogen donating ability [26]. The miswak extract showed a concentration dependent scavenging of DPPH radical, which may be attributed to its hydrogen donating ability (Figure 1). The DPPH assay IC_50_ value was found to be 4.8 μg/ml crude methanol extract. The correlation coefficient (R^2^) between crude methanol extract and DPPH scavenging activity was found to be 0.97 indicating the strong correlation. The high IC_50_ values were reported for medicinal plants *Spermaccoce exilis*, *S. articularis* and *Leea indica* (370, 65 and 25 μg crude extract, respectively) [27].

The Trolox equivalent antioxidant capacity assay was also used to evaluate free radical scavenging capacities of miswak. The assay is based on the ability of antioxidant to scavenge ABTS radicals. It is a simple and usually used method for the evaluation of antioxidant capacity [28,29]. The ABTS radical can be evaluated over a wide pH range, which is useful to study the effect of pH on antioxidant mechanism. Furthermore, the ABTS radical is soluble in water and organic solvents, enabling the determination of antioxidant capacity of both hydrophilic and lipophilic compounds/samples. The methanol extract of miswak showed a concentration dependent scavenging of ABTS radical (Figure 1). The ABTS assay IC_50_ value was found to be 1.6 μg crude methanol extract. The correlation coefficient (R^2^) between crude methanol extract and ABTS scavenging activity was found to be 0.99. Therefore, the miswak extract had three-fold free radical scavenging capacity for ABTS radical greater than DPPH radical.

**Figure 2:**
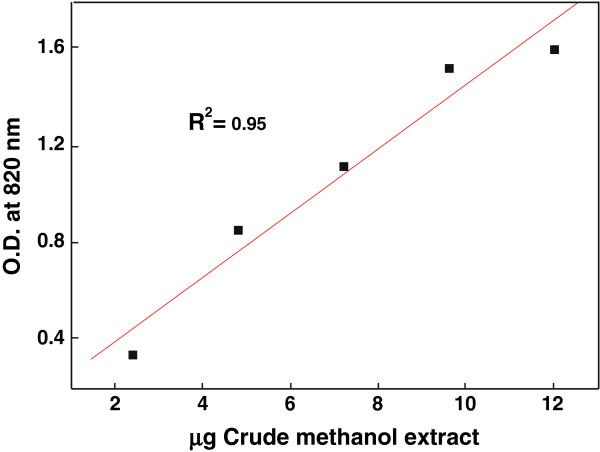
Correlation between different concentrations of miswak crude methanol extracts and their antioxidant capacity as determined by the formation of phosphomolybdenum complex assay.

The total antioxidant capacity is based on the reduction of molybdenum (VI) to molybdenum (V) by extracts and subsequent formation of a green phosphate/molybdenum (V) complex at acidic pH. The high absorbance values indicated that the sample possessed significant antioxidant activity. The methanol extract of miswak had significant total antioxidant activity and the effect increased with increasing concentration (Figure 2). The correlation coefficient (R^2^) between methanol extract of miswak and the formation of phosphomolybdenum complex was found to be 0.95. The optical density of 12 μg of methanol extract was 1.6. The similar optical density was detected for longan peel extracts at high concentrations ranged from 0.1 to 0.5 mg gallic acid equivalents [30].

**Figure 3:**
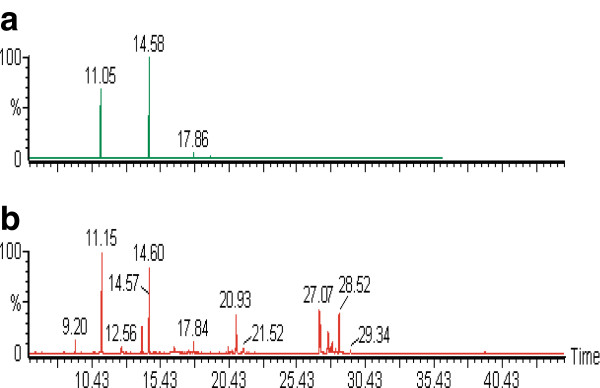
**GC-MS chromatograms of miswak crude methanol extract.** (**a**) without silylation, (**b**) with silylation.

The methanol extract of miswak was analyzed by GC-MS to determine its chemical composition. The GC-MS analysis showed that the methanol extract doesn’t contain phenolic and flavenoid compounds, which considered the main antioxidant compounds in plant, or under detected limit (Figure 3a). Howevere, *Pistacia lentiscus* exhibited good radical scavenging activity against DPPH, it showed a lack of flavonoids [31]. Therefore, we treated the methanol extract of miswak with trimethylsilyl chloride to derivatizate the hydroxyl and/or carboxylic functional groups that cause a problem in gas chromatographic separation. It involves the replacement of acidic hydrogen on the compound with an alkylsilyl group. After silylation of methanol extract, three compounds namely 2-furancarboxaldehyde-5-(hydroxymethyl), furan-2-carboxylic acid-3-methyl- trimethylsilyl ester and D-erythro-pentofuranose-2-deoxy-1,3,5-tris-O-(trimethylsilyl) were identified by GC-MS analysis (Figure 3b, Table 2). These furan derivatives as they contain hydroxyl groups could be possessed antioxidant activities. Very little information has been reported on the isolation of furan derivatives, with antioxidant activities, from plants. Osbeckic acid, a furan-carboxylic acid, was obtained from *Osbeckia chinensis* and shown to be an antioxidative synergist [32]. Five furan derivatives were isolated from leaves and twigs of *Scleropyrum pentandrum*. These compounds were evaluated for their radical scavenging activities using DPPH assay [33]. Recently, some synthetic furan derivatives were prepared and exhibited antioxidant activities [34,35].

**Table 2:** Chemical composition of furan derivatives detected by GC-MS in the miswak crude methanol extract treated with trimethylsilyl chloride

**Compound**	**Retention**	**Molecular**	**Formula**
	**time (min)**	**weight**	
2-Furancarboxaldehyde-5-(hydroxymethyl)	12.56	126	
Furan-2-carboxylic acid-3-methyl-, trimethylsilyl ester	14.06	198	
D-Erythro-pentofuranose-2-deoxy-1,3,5-tris-O-(trimethylsilyl)	27.17	290	

Generally, plant tissue antioxidant capacity is closely associated with the contents of antioxidant substances, mainly phenolic compounds, carotenoids, tocopherol and ascorbic acid and with activity of “free radical scavenging enzymes” (superoxide dismutase, catalase, peroxidase and polyphenoloxidase) [36]. Additionally, total phenolics and anthocyanins, as the main antioxidants, are involved in oxidative reactions, such as polyphenoloxidase and peroxidase. Therefore, peroxidase, catalase and polyphenol oxidase has been detected in miswak extract. Table 3 shows considerable value of peroxidase with low level of catalase and polyphenoloxidase. The biochemical properties of peroxidase from miswak were studied [37]. A Caribbean copper plant peroxidase from the latex of *Euphorbia cotinifolia* was studied [38]. Catalase and antioxidant activity were screened in nine medicinal plants traditionally used in Chinese medicine [39]. A partial characterization of polyphenoloxidase activity of herb *Thymus longicaulis subsp. chaubardii var. chaubardii* is described [40].

**Table 3:** The antioxidant enzyme activities in miswak

**Enzyme**	**Units/g tissues**
Peroxidase	4176 ± 150
Polyphenoloxidase	6.1 ± 0.2
Catalase	2.5 ± 0.04

Values are presented as means ± SE (n = 3).

## Conclusions

Furan derivatives, identified by GC-MS analysis, from miswak could be exhibited high antioxidant activity by scavenging DPPH radicals, ABTS radicals and reducing molybdenum (VI) to molybdenum (V). The antioxidant capacity of miswak was also attributed to the presence of antioxidant enzymes, peroxidase, caltalase and polyphenoloxidase. The synergistic actions of antioxidant compounds and antioxidant enzymes make miswak is a good chewing stick for cleaning teeth, oral hygiene and food purposes.

## Competing interests

Both authors declare that they have no competing interests.

## Authors’ contributions

MS and KJ performed all experiments and read and approved the final manuscript.

## Pre-publication history

The pre-publication history for this paper can be accessed here:

http://www.biomedcentral.com/1472-6882/13/40/prepub
